# Coordinated Changes in Antioxidative Enzymes Protect the Photosynthetic Machinery from Salinity Induced Oxidative Damage and Confer Salt Tolerance in an Extreme Halophyte *Salvadora persica* L.

**DOI:** 10.3389/fpls.2016.00050

**Published:** 2016-02-10

**Authors:** Jaykumar Rangani, Asish K. Parida, Ashok Panda, Asha Kumari

**Affiliations:** ^1^Division of Wasteland Research, Central Salt and Marine Chemicals Research Institute – Council of Scientific and Industrial ResearchBhavnagar, India; ^2^Academy of Scientific and Innovative Research, Central Salt and Marine Chemicals Research Institute – Council of Scientific and Industrial ResearchBhavnagar, India

**Keywords:** ascorbate peroxidase, catalase, halophyte, lipid peroxidation, photosynthesis, *Salvadora persica*, superoxide dismutase

## Abstract

Salinity-induced modulations in growth, photosynthetic pigments, relative water content (RWC), lipid peroxidation, photosynthesis, photosystem II efficiency, and changes in activity of various antioxidative enzymes were studied in the halophyte *Salvadora persica* treated with various levels of salinity (0, 250, 500, 750, and 1000 mM NaCl) to obtain an insight into the salt tolerance ability of this halophyte. Both fresh and dry biomass as well as leaf area (LA) declined at all levels of salinity whereas salinity caused an increase in leaf succulence. A gradual increase was observed in the Na^+^ content of leaf with increasing salt concentration up to 750 mM NaCl, but at higher salt concentration (1000 mM NaCl), the Na^+^ content surprisingly dropped down to the level of 250 mM NaCl. The chlorophyll and carotenoid contents of the leaf remained unaffected by salinity. The photosynthetic rate (P_N_), stomatal conductance (g_s_), the transpiration rate (E), quantum yield of PSII (ΦPSII), photochemical quenching (qP), and electron transport rate remained unchanged at low salinity (250 to 500 mM NaCl) whereas, significant reduction in these parameters were observed at high salinity (750 to 1000 mM NaCl). The RWC% and water use efficiency (WUE) of leaf remained unaffected by salinity. The salinity had no effect on maximum quantum efficiency of PS II (Fv/Fm) which indicates that PS II is not perturbed by salinity-induced oxidative damage. Analysis of the isoforms of antioxidative enzymes revealed that the leaves of *S. persica* have two isoforms each of Mn-SOD and Fe-SOD and one isoform of Cu-Zn SOD, three isoforms of POX, two isoforms of APX and one isoform of CAT. There was differential responses in activity and expression of different isoforms of various antioxidative enzymes. The malondialdehyde (MDA) content (a product of lipid peroxidation) of leaf remained unchanged in *S. persica* treated with various levels of salinity. Our results suggest that the absence of pigment degradation, the reduction of water loss, and the maintenance of WUE and protection of PSII from salinity-induced oxidative damage by the coordinated changes in antioxidative enzymes are important factors responsible for salt tolerance of *S. persica*.

## Introduction

Salinity is an important abiotic stress that reduces crop productivity worldwide. The effects of salinity on plants include reduced growth, ion toxicity, osmotic stress, mineral deficiencies, photosynthetic imbalance, and combinations of these effects (Jenkins et al., 2010; Galmés et al., 2011). High concentrations of salt have adverse effects on plant growth. The immediate response to salt stress is a reduction in the rate of leaf surface expansion as the salt concentration increases (Wang and Nil, 2000). An approximate 80% reduction in plant growth at high salinity is due to the reduction of leaf area (LA) expansion and the consequent reduction in light interception (Parida and Das, 2005). Approximately 20% of the growth reduction is most likely explained by a decrease in stomatal conductance (Parida and Das, 2005). One of the foremost effects of salinity is nutritional disorder that results from the effect of salinity on nutrient availability, competitive uptake and transport or partitioning within the plant. Salinity induces oxidative damage due to the overproduction of reactive oxygen species (ROS) such as superoxide ions ($O_{2}^{•-}$), hydrogen peroxide (H_2_O_2_), and hydroxyl radical (OH•) (Zheng et al., 2009). Uncoupling of the light and dark reactions due to the stomatal closure under saline conditions is the main cause of ROS production in chloroplasts (Ozgur et al., 2013). Antioxidative enzymes are indispensable components of ROS scavenging system. Plants contain a complex antioxidative defense system to eliminate ROS, which includes the low-molecular mass antioxidants such as carotenoids, ascorbate, and glutathione as well as ROS-scavenging enzymes such as superoxide dismutase (SOD), catalase (CAT), ascorbate peroxidase (APX), peroxidase (POX), and glutathione reductase (GR). SOD is a major scavenger of superoxide ($O_{2}^{•-}$), and its enzymatic action results in the formation of H_2_O_2_ and O_2_. The H_2_O_2_ produced is then scavenged by CAT and a variety of POXs (Parida et al., 2004b).

Different growth and development-related processes depend on the networking of intracellular organelles. The chloroplast is the site of photosynthesis where both light and dark reactions occur. However, this organelle is highly sensitive to stressful environments such as salinity, drought, etc. and therefore plays an important role in the monitoring of stress responses (Biswal et al., 2008). The regulation of leaf stomatal conductance (g_s_) is a key phenomenon in plants because it is essential for the prevention of desiccation and CO_2_ acquisition (Dodd, 2003; Medici et al., 2007; Ashraf and Harris, 2013). Decrease in leaf turgor pressure and atmospheric vapor pressure, cause stomatal closure under saline conditions (Chaves et al., 2009). The decrease in the photosynthetic rate is normally due to the suppression of mesophyll conductance and stomatal closure under moderate and severe stress conditions (Flexas et al., 2004; Chaves et al., 2009). Stomatal limitations for diffusion of gases alter photosynthesis and mesophyll metabolism (Parida et al., 2005; Chaves et al., 2009). Most importantly, salinity induces an ionic effects on organelle ultrastructure and the photosynthetic metabolic process (Sade et al., 2010). The high saline conditions negatively influence plant growth and development, inhibit photosynthesis (Sharma et al., 2005), cause water deficit (Suarez and Medina, 2008), and interfere with nutrition uptake, which leads to a nutrient imbalance.

The plant’s ability to acclimate to a saline environment includes characteristic changes in phyllotaxy that may be morphological, physiological, and biochemical. Many plants adjust to high salinity and consequently to low soil water availability (Ashraf, 2004). Halophytes differ from glycophytes in their tolerance to saline conditions (Shevyakova et al., 2006; Kachout et al., 2009). Halophytes have evolved a salt-tolerance mechanism that confers optimal growth under high saline conditions (Flowers and Colmer, 2008). The salt tolerance in halophytes is the result of an effective coordination of physiological and metabolic pathways (Kumari et al., 2015). Halophytes have a range of adaptations that not only helps them to adapt to but also to benefit from a saline environment (Shabala and Mackay, 2011). The salt tolerance mechanisms in halophytes include ion homeostasis, regulation of osmolarity by various osmolytes and, most importantly, antioxidative defense (Shabala, 2013; Shabala et al., 2014). In comparison to glycophytes, the halophytes possess unique anatomical structures that enable them to tolerate high salinity (Yu et al., 2011; Shabala, 2013). The salt glands, salt bladders, and salt hairs in the leaf of the halophytes excrete excess salt (Shabala, 2013). Another typical characteristic of the halophytes is the induction of leaf succulence by thickening of the leaves with increasing water content by which absorbed salt was diluted and salt-induced damage was reduced to some extent (Parida and Jha, 2010a). The development of kranz anatomy, dimorphism of chloroplast and the reduction in number of stomata per LA (stomatal density) are the typical characteristics in halophytes. The waxed epidermis in the leaves is a protective trait in some halophytes, which contributes to low transpiration of halophytes than glycophytes (Parida and Jha, 2010a; Shabala, 2013). The sequestration of Na^+^ into the vacuole is also an important strategy for osmotic adjustment and reduction of Na^+^ concentration in the cytosol of the halophytes (Shabala, 2013). Under saline conditions, halophytes tend to accumulate minerals such as Ca^+^ and Na^+^ in their vacuoles to use as an osmoticum. Halophytes avoid photo-damage by developing certain mechanisms to dissipate excess excitation energy (Nianwei et al., 2003). Stomatal closure under saline conditions helps to maintain leaf water content, but it decreases the CO_2_ assimilation rate (Parida et al., 2004a).

*Salvadora persica* L. (Miswak) is a desert facultative halophytic plant found to survive under very high saline conditions. *S. persica* belongs to the family *Salvadoraceae* and is a medium-sized tree. The plant contains several bioactive compounds such as alkaloids, tannins, saponins, and sterols that are used in the food and cosmetic industries (Sharma and Ramawat, 2013). *S. persica* is a facultative halophyte with wide adaptability ranging from deserts to heavy soil, non-saline to highly saline soil, and dry regions to marshy and waterlogged areas (Reddy et al., 2008). Many species require fresh water for their germination, but *S. persica* germinates in saline water of approximately 15 dS m^-1^ salt concentration (Rao et al., 2004). Therefore, an understanding of the salt tolerance ability of *S. persica* is imperative. Some preliminary works have been carried out on growth and mineral accumulation in *S. persica* treated with low salinity up to 200 mM NaCl for a short time (Maggio et al., 2000; Ramoliya et al., 2004). However, it can tolerate very extreme saline conditions. In the present investigation, detailed physiological and biochemical analyses have been carried out in the halophyte *S. persica* under long-term exposure to extreme salinity up to 1000 mM NaCl in green house conditions to obtain insight into the mechanisms of salt tolerance with a future aim to develop salt tolerant crops. Salinity-induced changes in growth, relative water content (RWC), mineral ions, photosynthesis, water use efficiency (WUE), photosynthetic pigments, and chlorophyll fluorescence parameters have been investigated in *S. persica* treated with various levels of salinity (0–1000 mM NaCl). The changes in the levels of various antioxidative enzymes and their isoforms have also been studied to elucidate the antioxidative defense mechanisms of *S. persica* to protect the plant from salinity-induced oxidative damage.

## Materials and Methods

### Plant Material, Growth Conditions, and Stress Treatment

Seeds of *S. persica* were collected from the CSMCRI salt farm area, Bhavnagar, Gujarat, India (latitude 21° 47.306′ N and longitude 72° 7.417′ E). The seeds were surface sterilized with 5% sodium hypochlorite for 30 min and washed three times with tap water and then with distilled water. The seeds were germinated in black plastic bags (W × L × H; 10 cm × 10 cm × 25 cm) containing soil, sand, and peat (2:1:1) under greenhouse conditions (50 ± 5% relative humidity, 1000–1250 μmole m^-2^ s^-1^ photosynthetic active radiance (PAR), 14 ± 2 h d^-1^ photoperiod from sunlight and 28 ± 5°C ambient temperature). The seedlings were irrigated with tap water every day. Two-month-old uniform-sized seedlings were transferred to plastic pots (W × H; 50 cm × 100 cm) containing the same soil mixture, acclimatized for 15 days, and then irrigated with Hoagland’s nutrient medium supplemented with various concentrations of NaCl (250, 500, 750, and 1000 mM). The control plants were grown in the nutrient medium devoid of NaCl. The plants were maintained in the greenhouse under the same environmental conditions as mentioned above. After 60 days of salt treatment, leaves from the same position of the plants were sampled from the control and the NaCl-treated plants for the measurement of various parameters.

### Morphological Observations, Measurement of Growth Parameters, and Leaf Succulence

Growth and morphological parameters such as plant height, LA, the number of branches, canopy coverage, and leaf succulence were measured in control and NaCl-treated plants. For measurements of fresh and dry weights of leaf stem and root, plant parts were excised from control and NaCl-treated plants, and the fresh weights were recorded immediately. Then, these plant parts were wrapped in pre-weighed aluminum foil and kept in an incubator at 70°C for 48 h before the dry weights were recorded. Total green LA per plant was measured in both control and NaCl-stressed plants using Digimizer software (version 4.3.1, MedCalc Software, Belgium). Leaf succulence, defined as the water content per unit area, was determined by taking 10 leaves from five plants from each treatment. The leaves were rinsed with distilled water and gently dried with tissue paper. Samples were dried for 48 h at 70°C to determine the dry mass (DM) after measuring the fresh mass (FM) and LA for each sample. The leaf succulence was estimated using the following equation:

$$Leaf\,\;succulence\,\;(mg\,\;{cm}^{-2})=(FM-DM)/LA.$$

### Determination of Mineral Ion Contents

Leaf samples were dried in an oven at 70°C for 48 h for analysis of various mineral ion contents. After drying, pre-weighed samples (approximately 0.5 g) were homogenized and placed in a 25 ml volumetric flask. Flasks containing the samples were placed on a hot plate at 350°C after adding 10 ml of an acidic mixture of HNO_3_ and HClO_4_ (9:4) in a fume hood and digested for 1 to 2 h until the production of red NO_2_ fumes ceased. The flask contents were further evaporated until the volume was reduced to 3–5 ml. Completion of digestion was confirmed when the liquid became colorless. After cooling the volumetric flasks to room temperature, 20 ml of deionized water was added and the volume was made up to 25 ml. The solution was filtered through Whatman No. 1 filter paper and stored. Aliquots of this solution were used for the determination of ions, *viz.*, Na^+^, K^+^, Ca^2+^, Mg^2+^, Fe^2+^, Mn^2+^, Zn^2+^, and Cu^2+^ by Inductively Coupled Plasma Atomic Absorption Spectrometry (Optima 2000DV, Perkin Elmer, USA). The total nitrogen content of the leaves was determined from dry leaf powder using an elemental analyzer (Elementar, Vario Micro Cube, Germany).

### Measurement of Photosynthesis and Chlorophyll Fluorescence Parameters

The net photosynthetic rate (P_N_), intercellular CO_2_ concentration (C_i_), stomatal conductance (g_s_), transpiration rate (E), and chlorophyll fluorescence were measured simultaneously using a leaf chamber fluorometer (6400-40 LCF, Li-Cor) attached to a LI-6400XT infrared gas analyzer (LI-COR^®^ Inc., Lincoln, NE, USA). Water-use efficiency (WUE) was calculated as the ratio between P_N_ and E (μmol CO_2_ assimilated per mol H_2_O transpired). Measurements were made on each plant in the five salinity treatments (*n* = 5). The sample chamber was set at a 500 μmol s^-1^ air-flow rate and a chamber temperature of 25°C with a light intensity of 1000 μmol m^-2^ s^-1^ PPFD. The photosynthetic and chlorophyll fluorescence measurements were taken between 10:00 and 12:00 h when the ambient light intensity was 1000–1200 μmol m^-2^ s^-1^.

### Measurement of Leaf Relative Water Content (RWC%)

The RWC of the leaves was measured using the method of Barrs and Weatherley (1962). The leaves were collected from control and NaCl-treated plants. The leaf fresh weight (LFW) was immediately measured after sampling, and then, the leaves were immersed in distilled water for 8 h at room temperature. The leaves were then blotted dry and the leaf turgid weight (LTW) was taken prior to incubation at 70°C for 48 h. After the incubation period, the leaf dry weight (LDW) was noted. The leaf RWC was calculated using the following formula:

RWC(%) =[(LFW−LDW)/(LTW−LDW)]×100

### *In vivo* Localization of ROS Such as H_2_O_2_ and $O_{2}^{•-}$ in Leaf Tissue

*In vivo* localization of H_2_O_2_ was carried out by histochemical staining using 3, 3′- diaminobenzidine tetra hydrochloride (DAB). The leaves harvested from control and NaCl-treated plants were vacuum infiltrated for 5 min into fresh DAB solution (1 mg/ml) prepared in 10 mM potassium phosphate buffer (pH 7.8) in 50 ml Falcon centrifuge tubes. Infiltration was carried out by building up a vacuum (100–150 mbar, for about 5 min) and releasing it two to three times until the leaves were completely infiltrated. The tubes containing the infiltrated leaves were kept in the dark overnight and thereafter placed under continuous light (300 μmole m^-2^ s^-1^) at 25°C for 8 h. The stained leaves were bleached in a warm destaining solution composed of methanol:acetic acid:glycerol (3:2:1). Bleaching of stained leaves was confirmed as the leaves became transparent. These chlorophyll-free leaves were fixed using a fixative reagent composed of methanol:deionized water:glycerol (5:4:1) and scanned using a scanner (V750 PRO, EPSON PERFECTION, USA).

*In vivo* detection of $O_{2}^{•-}$ was done by histochemical staining using nitrotetrazolium blue chloride (NBT). The leaves from control and NaCl-treated plants were vacuum infiltrated for 10 min into fresh NBT solution (1 mg/ml) prepared in 10 mM potassium phosphate buffer (pH 7.8) in 50 ml Falcon centrifuge tubes. The tubes were kept in the dark overnight and then exposed to continuous florescent light (300 μmole m^-2^ s^-1^) at 25°C for 8 h. The leaves were bleached in a hot methanol:acetic acid:glycerol (3:2:1) solution, the blue color was fixed with a fixative reagent composed of methanol:deionized water:glycerol (5:4:1), and the leaves were scanned using a scanner (V750 PRO, EPSON PERFECTION, USA).

The H_2_O_2_ and $O_{2}^{•-}$ detected in leaves were quantified and expressed in percentage of total LA from the scanned images of the leaves by calculating the area of the spots stained with DAB and NBT and total LA using Digimizer software (version 4.3.1, MedCalc Software, Belgium).

### Lipid Peroxidation

The degree of lipid peroxidation was measured by determining the concentration of malondialdehyde (MDA) produced by the thiobarbituric acid (TBA) reaction following the method of Draper and Hardley (1990). Pre-weighed leaf tissue was homogenized in 2 ml of 0.1% (w/v) TCA solution. The homogenate was centrifuged at 10,000 × *g* for 15 min at room temperature, the supernatant was collected, and 500 μl of the supernatant was mixed with 2 ml of 0.5% (w/v) TBA prepared in 20% (w/v) TCA. The mixture was incubated at 95°C for 30 min, and the reaction was stopped by placing the reaction tubes in an ice water bath. Samples were centrifuged at 10,000 × *g* for 5 min, and the absorbance of the supernatant was read at 532 nm. The concentration of MDA was calculated from the extinction coefficient of 155 mM^-1^ cm^-1^.

### Determination of Photosynthetic Pigments

Fresh leaves (0.1 g) were thoroughly homogenized in 2 ml of pre-chilled 100% *N*, *N*-dimethylformamide (DMF) using a mortar and pestle in the dark at 4°C, and the homogenate was centrifuged at 15,000 × *g* for 15 min. The supernatant was collected and diluted fivefold. The absorbance at 664, 647, and 461 nm was recorded using a microplate reader (Epoch^TM^, BioTek, USA). Chlorophyll a (Chl a), chlorophyll b (Chl b) and total chlorophyll contents were estimated using the equations of Inskeep and Bloom (1985). The carotenoid contents were estimated using the equation of Chamovitz et al. (1993).

### Extraction and Measurement of the Activity of Various Antioxidant Enzymes

Shoot tissue (0.5 g) was ground to a fine powder in liquid N_2_ and then homogenized in 2 ml of 50 mM potassium phosphate buffer (pH 7.0), 1 mM EDTA, 0.05% (w/v) Triton X-100, and 5% (w/v) polyvinylpolypyrrolidone (PVPP) using a chilled pestle and mortar. The homogenate was centrifuged at 15,000 × *g* for 15 min at 4°C, and the supernatant was collected and used for the assays of CAT, guaiacol POX, GR, and SOD. For APX activity, a separate extraction was done using the buffer mentioned above except that it contained an additional 5 mM ascorbate to protect the APX activity. The protein concentrations in the enzyme extract was determined by the method of Bradford (1976).

### Superoxide Dismutase (EC 1.15.1.1)

Superoxide dismutase activity was measured by its ability to inhibit the photoreduction of nitroblue tetrazolium (NBT) as described by Parida and Jha (2010b). The reaction mixture (2 ml) contained 50 mM KPO_4_ (pH 7.8), 9.9 mM L-methionine, 58 μM NBT, 0.025% Triton X-100, 2.4 μM riboflavin, and 50 μl of enzyme extract.

### Catalase (EC 1.11.1.6)

Catalase activity was determined spectrophotometrically by measuring the initial linear rate of the decrease in absorbance at 240 nm due to the disappearance of H_2_O_2_ as described by Parida and Jha (2010b) using an extinction coefficient (ε) at 240 nm of 43.6 M^-1^ cm^-1^. The reaction mixture contained 50 mM potassium phosphate (pH 7.0) and 10.5 mM H_2_O_2_.

### Ascorbate Peroxidase (EC 1.11.1.11)

Ascorbate peroxidase was assayed as described earlier by Parida and Jha (2010b). The reaction mixture contained 50 mM potassium phosphate (pH 7.0), 0.5 mM ascorbic acid, and 0.1 mM H_2_O_2_. The decrease in absorbance at 290 nm for 1 min was recorded, and the amount of ascorbate oxidized was calculated from the extinction coefficient 2.8 mM^-1^ cm^-1^.

### Guaiacol Peroxidase (EC 1.11.17)

Guaiacol POX activity was measured spectrophotometrically at 25°C by following the method described by Parida and Jha (2010b). The reaction mixture (2 ml) consisted of 50 mM potassium phosphate (pH 7.0), 9 mM guaiacol, and 19 mM H_2_O_2_. The formation of tetraguaiacol was measured at 470 nm (ε = 26.6 mM^-1^ cm^-1^).

### Glutathione Reductase (EC 1.6.4.2)

Glutathione reductase activity was estimated by measuring the rate of reduction of 5, 5′-dithiobis-(2-nitrobenzoic acid) (DTNB) as an increase in absorbance at 412 nm (ε = 14.15 mM^-1^ cm^-^1) according to the procedure described in Parida and Jha (2010b). The reaction mixture (1 ml) consisted of 100 mM KPO_4_ (pH 7.5), 1 mM EDTA, 1 mM oxidized glutathione (GSSG), 0.75 mM DTNB, 0.1 mM NADPH, and the enzyme.

One unit of SOD was defined as the amount of enzyme that inhibited 50% of the NBT photoreduction. For CAT, APX, POX, and GR, one unit of enzyme was defined as the amount of enzyme necessary to decompose 1 μmol of substrate per minute at 25°C.

### Native PAGE and Activity Staining of Various Antioxidant Enzymes

Native polyacrylamide gel electrophoresis (PAGE) was performed at 4°C for SOD, CAT, POX, APX, and GR. Samples were mixed with 20% glycerol (v/v) and 0.25% bromophenol blue before loading onto the gels. An equal amount of protein (20 μg) was loaded in each lane. The gels were run at a constant 200 V at 4°C in a Bio-Rad Mini protein electrophoresis system. Isoforms of SOD were resolved in a 10% native polyacrylamide gel and visualized by NBT staining (Beauchamp and Fridovich, 1971). The different isoforms of SOD were identified by selective inhibition with H_2_O_2_ and potassium cyanide (KCN) following the method described earlier by Miszalski et al. (1998). The isoforms of Cu/Zn SOD and Fe-SOD were inhibited by staining the gel in solution containing 5 mM H_2_O_2_ and selective inhibition of Cu/Zn SOD was carried out by incubating the gel in solution containing 3 mM KCN. CAT isoforms were separated in a 7.5% native polyacrylamide gel and were visualized by staining with 0.03% (v/v) H_2_O_2_, 1% ferric chloride, and 1% potassium ferricyanide following the method of Woodbury et al. (1971). APX isoforms were separated in a 10% native polyacrylamide gel and were stained following the method of Mittler and Zilinskas (1993). Isoforms of POX were resolved in a 7.5% polyacrylamide gel and visualized by staining with a solution containing 50 mM sodium acetate buffer (pH 5.4), 10 mM *O*-dianisidine and 10 mM H_2_O_2_. GR isoforms were separated in a 7.5% native polyacrylamide gel and detected by incubating the gels in 50 mM KPO_4_ (pH 7.5) containing 0.24 mM monotetrazolium, 0.34 mM 2,6-dichlorophenolindophenol, 3.4 mM oxidized glutathione (GSSG) and 0.5 mM NADPH. The gels were scanned and analyzed using a scanner (V750 PRO, EPSON PERFECTION, USA).

### Statistical Analysis

All the experiments were conducted with a minimum of six replicates, and the results were expressed as the mean ± standard deviation (SD). All the data were subjected to one-way analysis of variance (ANOVA) and Duncan’s multiple-range test (*P* ≤ 0.05) using the Sigma Plot v12.0 statistical software (Systat Software Inc., Chicago, IL, USA).

## Results

### Effects of Salinity on Plant Morphology and Growth

**FIGURE 1:**
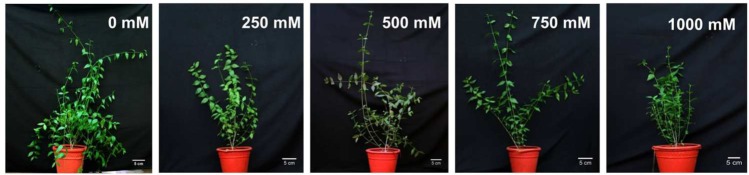
**Morphological changes in *Salvadora persica* seedlings after 60 days of treatment with various levels of NaCl**.

**FIGURE 2:**
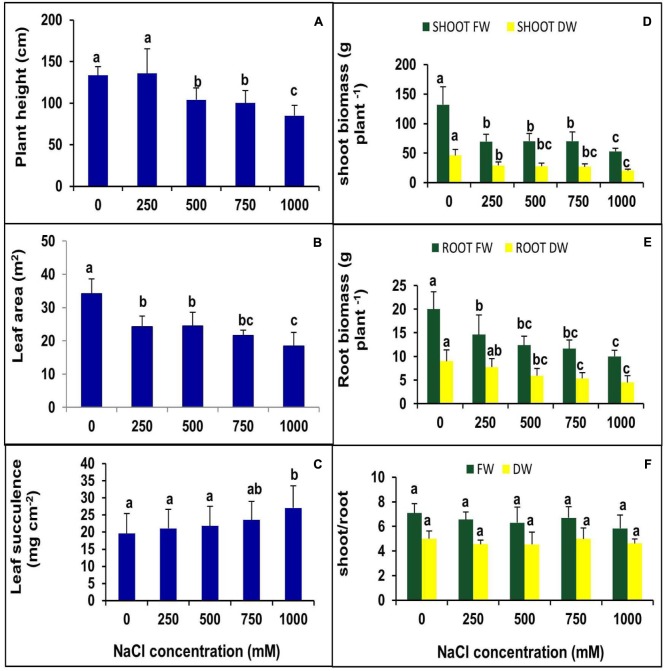
**Effects of various levels of NaCl on **(A)** plant height; **(B)** leaf succulence; **(C)** leaf area (LA); **(D)** shoot biomass; **(E)** root biomass; **(F)** shoot/root ratio of *S. persica* seedlings.** The values are mean ± SD (*n* = 6). The different letters on the top of the error bars indicate statistically different means at *P* ≤ 0.05.

The effects of NaCl on plant morphology were analyzed by plant growth, LA, the number of branches and canopy coverage after 90 days of salt treatment. The lower concentration of NaCl (250 mM) treatments showed no negative effects on growth, but at higher concentrations, plant growth decreased significantly (**Figure 1**). Canopy, as well as the number of branches, were reduced with increasing salt concentration (**Figure 1**). Plant height was not changed significantly at the 250 mM NaCl treatment as compared to control but gradually decreased with increasing NaCl concentration. There was no significant change was observed in plant height between 500 and 750 mM NaCl treated plants, whereas it declined by 21% in the 1000 mM NaCl treated plants as compared to control (**Figure 2A**). Salinity induced a reduction in leaf size, but the thickness of leaves increased with increasing salinity. The total LA per plant decreased in salt-treated plants compared to the control, but no significant change was observed among the treatments (**Figure 2B**). The leaf succulence in *S. persica* showed no significant change at moderate or high salinity, but at the extreme salt concentration (1000 mM NaCl), succulence increased significantly to 27.04 mg cm^-2^ from 19.59 mg cm^-2^ in the control plants (**Figure 2C**). The fresh leaf, stem, and root biomass decreased with increasing salinity. Total shoot biomass decreased with increasing salt concentration as compared to the control, but the change was not significant for the 250, 500, and 750 mM NaCl salt treatments. However at extreme salinity (1000 mM NaCl), the total shoot biomass decreased significantly (**Figure 2D**). The root growth decreased in 250 mM NaCl concentration compared to the control, but at higher salt concentrations (500 and 750 mM NaCl), no significant changes were observed with respect to each other (**Figure 2E**). There was no significant change observed in shoot to root ratio in any salt concentration compared to the control (**Figure 2F**).

### Effects of Salinity on RWC%

**FIGURE 3:**
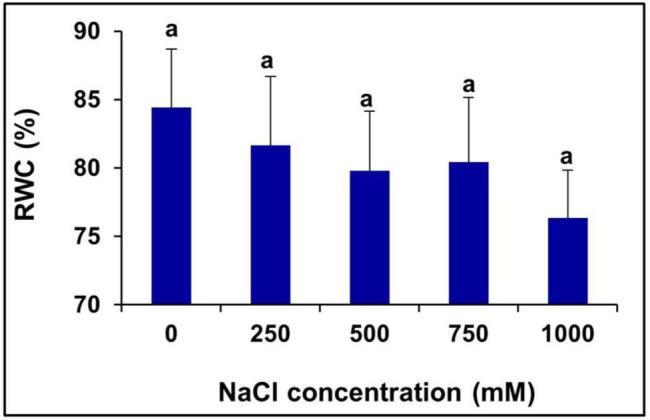
**Salinity induced changes in the leaf RWC % of *S. persica*.** The values are mean ± SD (*n* = 6). The different letters on the top of the error bars indicate statistically different means at *P* ≤ 0.05.

There was no significant change observed in the leaf RWC (%) of the plants treated with various levels of salinity (**Figure 3**).

### Salinity Induced Accumulation of ROS Such as H_2_O_2_ and $O_{2}^{•-}$

**FIGURE 4:**
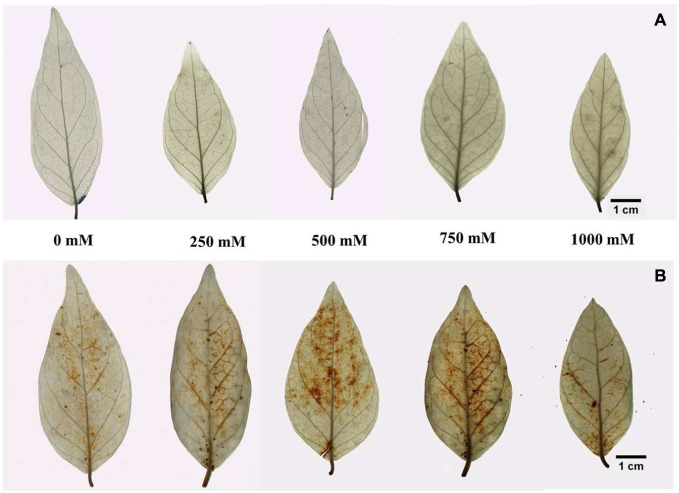
***In vivo* localization of ROS in the leaf tissue of *S. persica* seedlings treated with various levels of salinity **(A)** Localization of superoxide by histochemical staining with nitro blue tetrazolium (NBT) and **(B)** localization of H_2_O_2_ by histochemical staining with 3, 3- diaminobenzidine tetra hydrochloride (DAB)**.

In our study, it was noticed that there was no significant accumulation of H_2_O_2_ and $O_{2}^{•-}$ in leaf tissue with increasing salinity compared to the control. There was a slight accumulation of H_2_O_2_ observed both in control and treated samples, but the level of $O_{2}^{•-}$ observed in the leaf lamina of the control and treated plants were very low (**Figure 4**). The accumulation of superoxide radicals was observed only in the injured parts of the petiole (**Figure 4**). The level of H_2_O_2_ and $O_{2}^{•-}$ quantified in terms of percentage of total LA was found to be 4.8 to 7.7% and 0.02 to 0.35%, respectively.

### Effects of Salinity on Lipid Peroxidation

**FIGURE 5:**
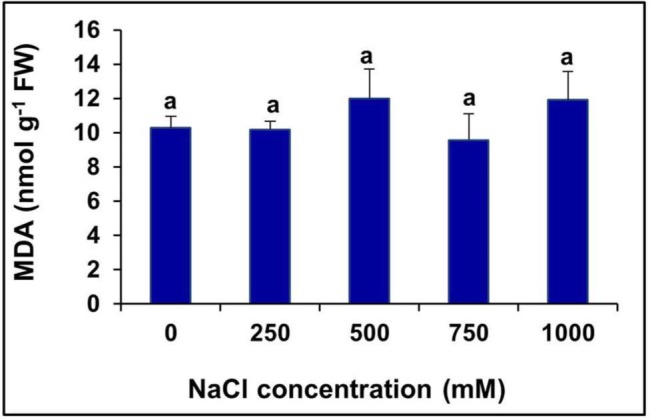
**Changes in lipid peroxidation level measured in terms of MDA content in the in the leaves of *S. persica* seedlings treated with various salt concentrations.** The values are mean ± SD (*n* = 6). Means followed by different letters are significantly different at *P* ≤ 0.05. The values are mean ± SD (*n* = 6). The different letters on the top of the error bars indicate statistically different means at *P* ≤ 0.05.

The level of MDA, a product of lipid peroxidation, remained unchanged at all levels of salinity, even in the extreme salt treatment (1000 mM NaCl), compared to the control (**Figure 5**).

### Effects of Salinity on Mineral Ion Content

The Na^+^ content in the leaves of salt-treated seedlings of *S. persica* gradually increased with increasing salt concentration up to 750 mM NaCl concentration, but at 1000 mM NaCl concentration, it dropped down to the level of 250 mM NaCl treatment. The Na^+^ content was increased by 39.22, 77.38, 90.76, and 39.41% in 250, 500, 750, and 1000 mM NaCl treated plants, respectively, compared to the control (**Table 1**). There was no significant changes in the K^+^ content at all levels of salinity in comparison to the control, but the Mg^2+^ content was reduced significantly at 1000 mM NaCl concentration. There was no significant changes observed in the Ca^2+^ content up to the 750 mM NaCl, but it was almost reduced by half in 1000 mM NaCl treated plants with respect to the control (**Table 1**). Minor elements were not momentously affected by salt stress except for Cu^2+^. The Cu^2+^ content decreased gradually with increasing salt concentration, and the maximum decrease of 60% was observed in 1000 mM NaCl treated plants (**Table 1**). The nitrogen content was increased in 250 mM NaCl treatment by 30% and then decreased by 32% at extreme salinity as to the control. However, the level of nitrogen was statistically at the control level in 500 and 750 NaCl treated plants.

**Table 1:** Effects of salinity on the contents of macro and micro nutrients in the leaves of *Salvadora persica*.

NaCl (mM)	Na^+^ (mg g^-1^ DW)	K^+^ (mg g^-1^ DW)	Ca^2+^ (mg g^-1^ DW)	Mg^2+^ (mg g^-1^ DW)	*N* (mg g^-1^ DW)	Fe^2+^ (μg g^-1^ DW)	Mn^2+^ (μg g^-1^ DW)	Zn^2+^ (μg g^-1^ DW)	Cu^2+^ (μg g^-1^ DW)
0	22.19 ± 3.6a	10.25 ± 2.0a	62.16 ± 2.5a	5.40 ± 1.3a	13.00 ± 1.8b	86.90 ± 25.1a	76.56 ± 9.3ab	36.05 ± 11.1a	39.88 ± 19.8a
250	30.89 ± 4.2b	9.19 ± 0.5ab	57.85 ± 1.8a	5.13 ± 1.1ab	16.93 ± 1.4a	56.12 ± 19.5b	67.19 ± 19.9a	26.26 ± 4.1a	22.10 ± 9.1b
500	39.36 ± 5.1c	7.79 ± 0.6ab	53.43 ± 6.0a	6.01 ± 0.7a	11.93 ± 1.5bc	66.42 ± 19.6ab	92.63 ± 23.1ab	33.50 ± 8.8a	21.17 ± 7.0b
750	42.33 ± 11.0c	10.30 ± 0.8a	66.18 ± 10.9a	6.74 ± 1.3a	11.52 ± 1.5bc	73.76 ± 25.0ab	99.29 ± 21.7b	35.56 ± 5.8a	16.96 ± 6.9b
1000	30.93 ± 3.7b	8.91 ± 1.7ab	29.61 ± 3.5b	3.84 ± 0.8b	8.97 ± 1.5c	76.33 ± 12.2ab	66.67 ± 21.3a	37.44 ± 9.8a	15.79 ± 4.4b


*The values are mean ± SD (*n* = 6). Means followed by different letters are significantly different at *P* ≤ 0.05.*

### Effects of Salinity on the Pigment Content

**FIGURE 6:**
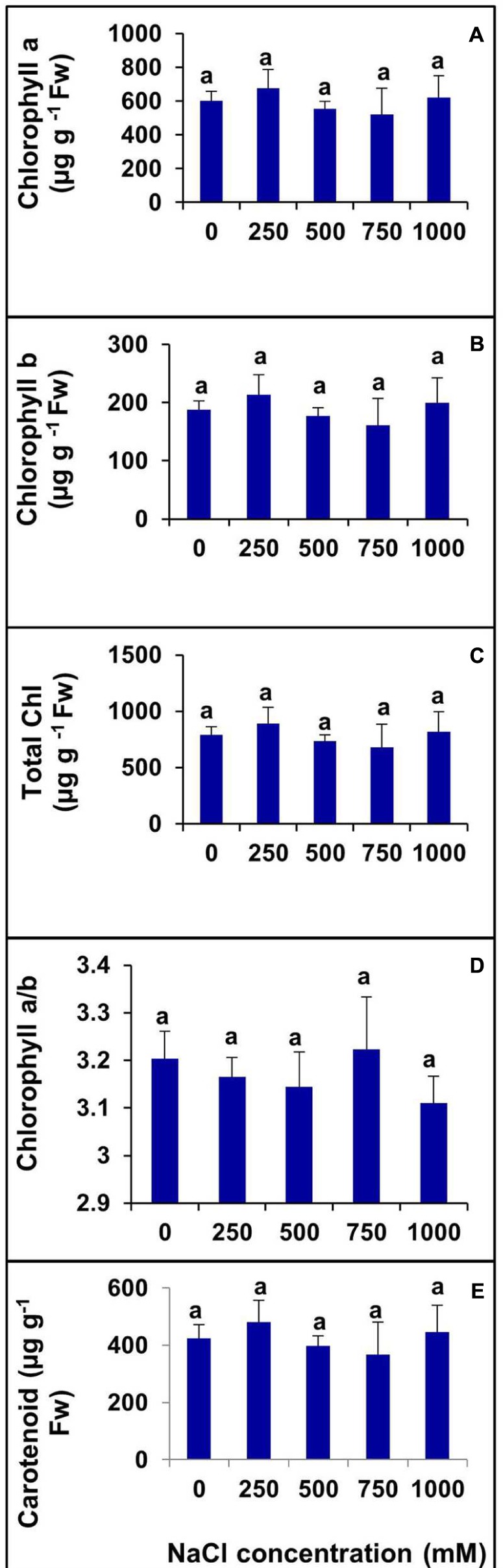
**Changes in photosynthetic pigments in leaf of *S. persica* seedlings treated with various levels of salinity.** **(A)** Chlorophyll a; **(B)** Chlorophyll b; **(C)** Total Chlorophyll; **(D)** Chl a/b; and **(E)** Carotenoid. The values are mean ± SD (*n* = 6). The different letters on the top of the error bars indicate statistically different means at *P* ≤ 0.05.

Various photosynthetic pigments were investigated in the halophyte *S. Persica* grown under various salt treatments for 60 days. There was no significant changes were evident in chlorophyll a, chlorophyll b, total chlorophyll, and carotenoid contents with increasing salinity (**Figures 6A–C,E**). The chlorophyll a/b ratio also remained unchanged in the control and treated plants (**Figure 6D**).

### Effects of Salinity on Photosynthetic Parameters

**FIGURE 7:**
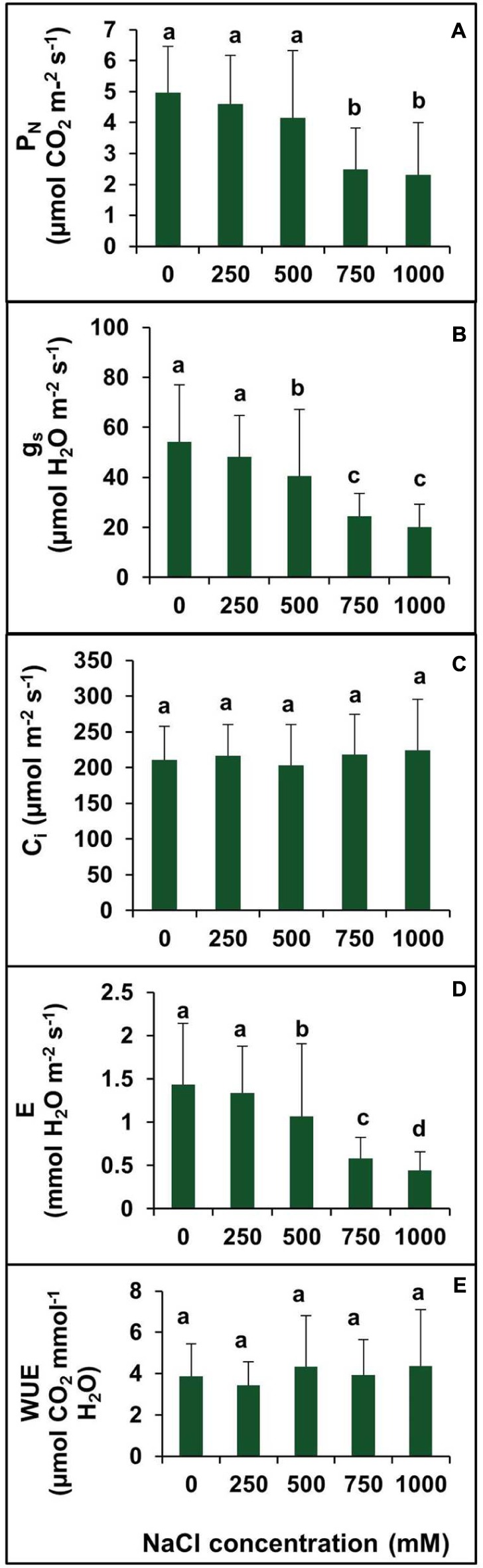
**Effect of various levels of salinity on photosynthetic parameters of *S. persica* seedlings.** **(A)** Net photosynthetic rate (P_N_); **(B)** stomatal conductance (g_s_); **(C)** intercellular CO_2_ concentration (C_i_); **(D)** transpiration rate **(E)**, and **(E)** water use efficiency (WUE). The values are mean ± SD (*n* = 6). The different letters on the top of the error bars indicate statistically different means at *P* ≤ 0.05.

The net photosynthetic rate (P_N_) remained unchanged in plants treated with salinity from 0 to 500 mM NaCl (**Figure 7A**). However, P_N_ decreased significantly at 750 and 1000 mM NaCl by 49.79 and 53.42%, respectively, with respect to the control (**Figure 7A**).There was no significant changes observed in *g*_s_ between the control and 250 mM NaCl treated plants. In contraststs, a decrease in the *g*_s_ value was observed in higher salt treatments (500–750 mM). The maximum decrease in the *g*_s_ was observed at salinity levels of 750 and 1000 mM which was by 2.2- and 2.7-folds, respectively, as compared to control (**Figure 7B**). Moreover, the intercellular CO_2_ concentration (*C*_i_) remained unchanged at all levels of salinity (**Figure 7C**). The *C*_i_ under salt stress peaked at 249.7 μmol mol^-1^ for 500 mM NaCl concentration. The transpiration rate (*E*) was in accordance with the stomatal conductance. The rate of transpiration was not significantly changed at lower salinity, but with an increase in salinity, the transpiration rate decreased significantly. At lower salt concentration (250 mM), the transpiration rate was identical to control value, but at high and extreme salinity, the transpiration rate was decreased by 28.5, 59.2, and 68.5% in 500, 750, and 1000 mm NaCl treated plants, respectively, as compared to control (**Figure 7D**). There was no apparent change in WUE observed in plants treated with various levels of salinity (0–1000 mM NaCl (**Figure 7E**).

### Effects of Salinity on Chlorophyll Fluorescence Parameters

**FIGURE 8:**
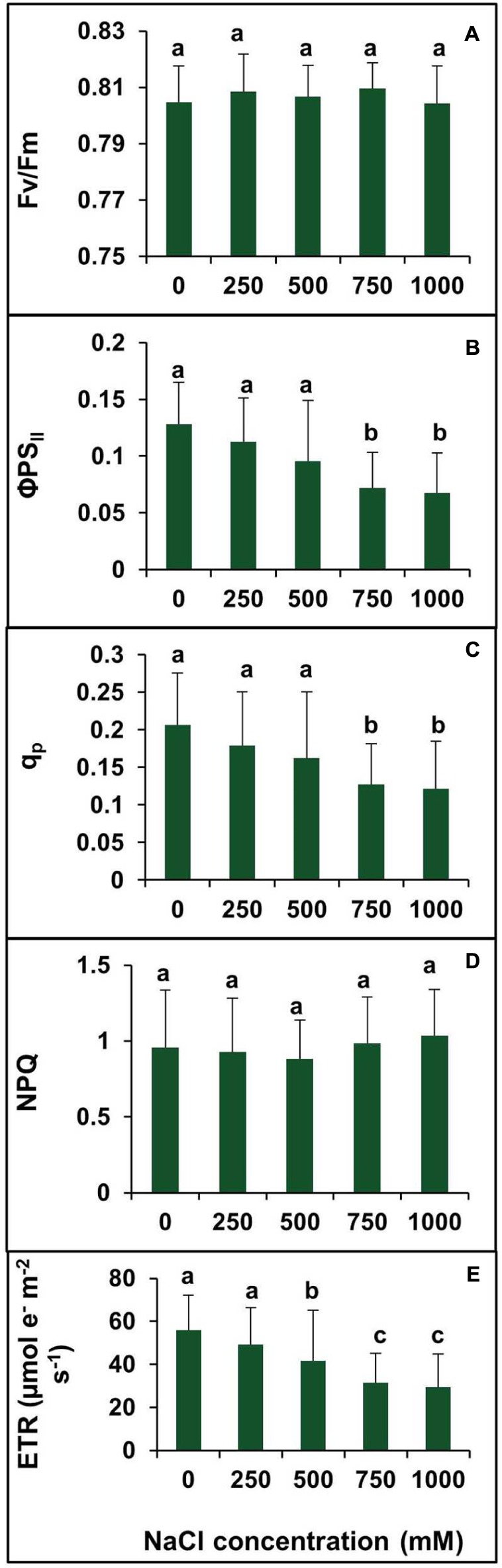
**Effects of various levels of salinity on chlorophyll fluorescence parameters of *S. persica* seedlings.** **(A)** Maximum quantum efficiency of PSII (Fv/Fm); **(B)** quantum yield of PSII (ΦPSII); **(C)** photochemical quenching (qP); **(D)** non-photochemical quenching (NPQ); and **(E)** electron transport rate (ETR). The different letters on the top of the error bars indicate statistically different means at *P* ≤ 0.05.

To investigate the changes in PSII photochemistry, photoinhibition, utilization, and dissipation of excess excitation energy in salt-adapted *S. persica*, measurements of various fluorescence parameters related to PSII photochemistry were analyzed for both the control and treated plants. The maximum efficiency of PSII (Fv/Fm) was measured to see whether the sensitivity of the plants to photoinhibition was increased. **Figure 8A** showed no significant difference in the Fv/Fm between the control and salt treated *S. persica* seedlings. **Figure 8B** depicts the efficiency of photosystem II (ΦPS_II_), which did not change apparently up to 500 mM NaCl treatment but decreased significantly at high (750 mM NaCl) and extreme (1000 mM NaCl) salinities. ΦPS_II_ was decreased by 41.6 and 47.2% in 750 and 1000 mM NaCl treated plants, respectively, compared with the control (**Figure 8B**). The photochemical quenching (*q*_p_) showed similar trend like ΦPS_II_ (**Figure 8C**). The value of *q*_p_ was not affected significantly at the lower salinities (250–500 mM NaCl) treatments. However, *q*_p_ was decreased by 38.3 and 41.2%, respectively, in 750 mM and 1000 mM NaCl treated plants as compared to control. There was no considerable change in non-photochemical quenching (NPQ) at all levels of salinity (**Figure 8D**). **Figure 8E** shows the changes in electron transport rate (ETR) in plants treated with various salt concentrations. The value of ETR remained unchanged in control and 250 mM NaCl treated plants and then declined at higher salt concentrations. As evidenced from our data, ETR decreased by 25.7, 44, and 47.5%, respectively, in 500, 750, and 1000 mM NaCl treated plants in comparision to control.

### Effects of Salinity on Antioxidant Enzymes

**FIGURE 9:**
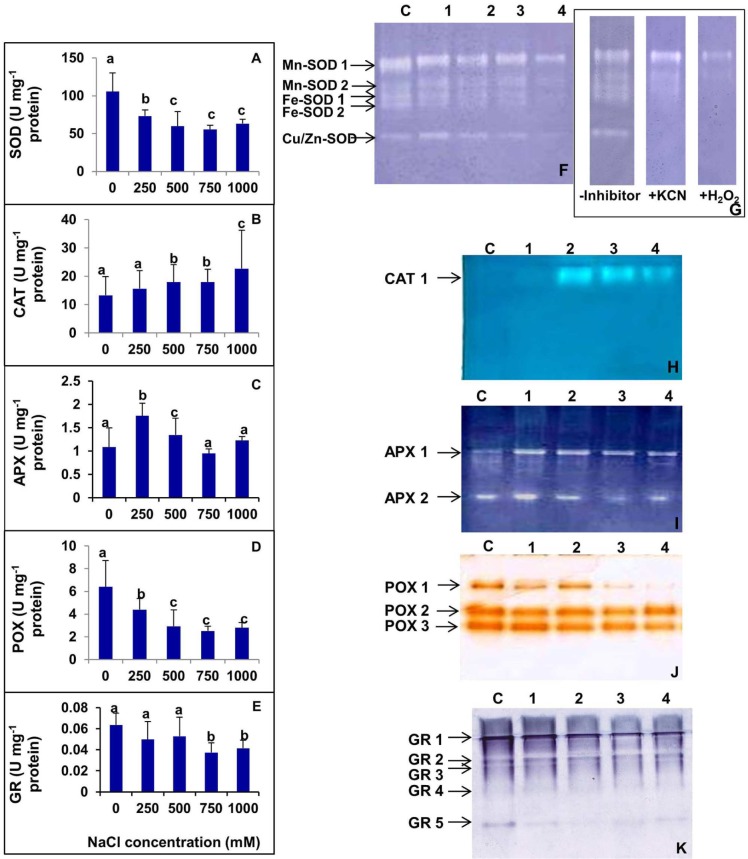
**(A–E)** Salinity induced changes in activity of various antioxidative enzymes in leaf of *S. persica* seedlings. **(A)** Superoxide dismutase (SOD); **(B)** Catalase (CAT); **(C)** ascorbate peroxidase (APX); **(D)** peroxidase (POX), and **(E)** glutathione reductase (GR). **(F–K)** Salinity induced changes in isoforms of various antioxidative enzymes in leaf of *S. persica* seedlings as analyzed by native PAGE. **(F)** Superoxide dismutase (SOD); **(G)** Identification of SOD isoforms in the leaves. Staining for activity was performed without any inhibitor, in the presence of 3 mM KCN that inhibits Cu/Zn-SOD, or in the presence of 5 mM H_2_O_2_, which inhibits both Cu/Zn- and Fe-SOD; **(H)** CAT; **(I)** APX; **(J)** POX; and **(K)** GR. Lane C-control; lane 1- 250 mM; lane 2- 500 mM; lane 3- 750 mM; and lane 4-1000 mM NaCl treated samples. The values are mean ± SD (*n* = 6). The different letters on the top of the error bars indicate statistically different means at *P* ≤ 0.05.

The activities of various antioxidant enzymes such as SOD, CAT, APX, POX, and GR were analyzed in *S. persica* (**Figure 9**). **Figure 9A** shows the changes in SOD activity after exposure to various concentrations of NaCl. The SOD activity decreased with increasing external salinity. A constitutively high level of SOD (105.8 Umg^-1^ protein) was observed in the control plants. According to our observations, SOD activity was declined by 30.54, 43.41, 47.41, and 40.31%, respectively, in 250, 500, 750, and 1000 mM NaCl treated plants as compared to control. The changes in SOD activity was not significant among 500, 750, and 1000 mM NaCl treatments (**Figure 9A**). Analysis of the isoforms of the antioxidative enzymes revealed that the leaves of *S. persica* have two isoforms each of Mn-SOD and Fe-SOD and one isoform of Cu/Zn-SOD (**Figures 9F,G**). The levels of all the isoforms of SOD was constitutively high in the control compared to high salt treated plants. It was also observed that the expression of Mn-SOD 1 was high at all levels of salinity as compared to other isoforms of SOD (**Figures 9F,G**).

As shown in **Figure 9B**, the activity of CAT did not change significantly as compared between control and the plants treated with low salt (250 mM NaCl). However, the CAT activity increased by 34.5% both in 500 and 750 mM NaCl treated plants and 70.6% in 1000 mM NaCl treated plants as compared to control (**Figure 9B**). The native PAGE and activity staining of CAT showed the presence of a single isoform of CAT (**Figure 9H**). The expression of CAT isoform increased in high salt treated plants (500–1000 mM NaCl; **Figure 9H**).

The activity of APX was increased significantly by 62.1 and 19.4% respectively in 250 and 500 mM NaCl treated plants compared to the control (**Figure 9C**). However, the extent of increase was not significant in plants treated with higher salinity (750–1000 mM NaCl). The APX level was declined to the control level at higher salt concentrations (**Figure 9C**). Activity staining of APX showed two isoforms in a native PAGE (**Figure 9I**). The level of APX-1 was preferentially enhanced in salt treated plants as compared to control. However, the level of isoform APX-2 remained apparently same in the control and NaCl treated samples.

High levels of POX were observed in control *S. persica* with respect to the treated plants. The POX activity gradually decreased with increasing salt treatments. POX activity was decreased by 31.29, 54.30, 60.71, and 56.49%, respectively, in 250, 500, 750, and 1000 mM NaCl treated plants as compared to control (**Figure 9D**). Activity staining showed three isoforms of POX as visualized in a native gel. The expression of all the isoforms of POX decreased gradually with increasing salinity. Among all the POX isoforms, the most prominent decrease was observed in POX 1 (**Figure 9J**).

As evidenced from **Figure 9E**, there was no significant changes in GR activity in plants treated with salinity up to 500 mM NaCl. However, GR activity was deceased significantly by 38.3 and 31.6%, respectively, in plants treated with 750 and 1000 mM NaCl in respect to the control. Activity staining specific for GR showed five isoforms in a native gel (**Figure 9K**). The band intensity of GR-1 and GR-5 isoforms decreased significantly with increasing salinity. However, there was no apparent changes in intensities of GR-2, GR-3, and GR-4 isoforms in control and salt treated samples (**Figure 9K**).

## Discussion

The glycophytes as well as halophytes experience oxidative stress when imposed to high salinity due to ionic and osmotic imbalance of the cell (Hasegawa et al., 2000; Lokhande et al., 2011). In halophytes, the major contributors for maintaining the cellular osmotic potential are Na^+^ and Cl^-^ (Flowers et al., 2015). The halophytes accommodates high concentrations of Na^+^ and Cl^-^ in tissues by intracellular compartmentation and the synthesis of compatible solutes. The bulk of the ions are compartmentalized within vacuoles and organic solutes such as sucrose, sugar alcohols, proline, and glycinebetaine are accumulated by halophytes and most probably contribute to osmotic adjustment in the cytoplasmic compartments of vacuolated cells rather than the whole cell (Flowers and Colmer, 2008; Flowers et al., 2015). The successful sequestration of Na^+^ and Cl^-^ into the vacuoles requires tonoplast-located ion exchangers and the H^+^ pumps that generate the electrochemical difference of H^+^ across the tonoplast to drive them; the H^+^ pumps also contribute to the membrane potential, which in turn influences channel transport activity (Munns and Tester, 2008; Hasegawa, 2013; Shabala, 2013; Flowers et al., 2015). The salt bladders which are found in about 50% of all the halophyte species are the most remarkable anatomical feature of halophytes (Flowers and Colmer, 2008; Shabala, 2013). The epidermal bladder cells (EBCs) which are 10 times bigger than the epidermal cells, represent a good possibility to sequester excessive Na^+^ away from metabolically important mesophyll cells of the leaf (Shabala, 2013). It has been reported that each EBC could sequester 1000-fold more Na^+^ as compared to epidermal cells (Shabala and Mackay, 2011; Shabala, 2013). The halophytes dependent on greatly on the use of inorganic ions (Na^+^, Cl^-^, and K^+^) to maintain shoot osmotic and turgor pressure under saline conditions, while glycophytes achieve this predominantly by increased *de novo* synthesis of compatible solutes (Shabala, 2013). The three major inorganic ions, Na^+^, K^+^ and Cl^-^, constitutes 80–95% of the osmotic pressure of the cell sap in halophytes, while in non-halophyte species the contribution is between 50 to 70% (Shabala and Mackay, 2011; Shabala, 2013).

The growth of halophytes is stimulated under moderate salinity conditions (Riadh et al., 2010). In the present investigation with *S. persica*, it was observed that there was no significant changes in growth between the control and the plants treated with 250 mM NaCl, suggesting that this level of salinity is within the threshold level for the growth of *S.* persica. There are several reports of stimulation of growth of halophytes at low salinity (Parida and Jha, 2010b; Redondo-Gómez et al., 2010; Takagi and Yamada, 2013). Although, there was a significant reduction in growth of *S. persica* plants treated with higher salinity (500, 750, and 1000 mM), but the plants survived up to 60 days without any visible symptoms of leaf wilting even in extreme salinity condition (1000 mM). However, salt tolerance limit of several halophytes are reported to be very lower than *S. persica*. Most of the halophytic plants such as *Bruguiera parviflora* (Parida et al., 2004a), *Suaeda salsa* (Qiu-Fang et al., 2005; Takagi and Yamada, 2013), *Salicornia branchiata* (Parida and Jha, 2010b), *Hordeum marinum* (Seckin et al., 2010), *Gypsophila oblanceolata* (Sekmen et al., 2012), and *Acacia ampliceps* (Theerawitaya et al., 2015) cannot thrive for long days at salt concentrations of beyond 600 mM. In contrasts to halophytes, the salt tolerance limit of most of the glycophytic plants are in the range of 50–250 mM (Hernández et al., 2000; Kholova et al., 2009; D’Souza and Devaraj, 2010; Nazar et al., 2011; Akçay et al., 2012; Boriboonkaset et al., 2013; Parida and Jha, 2013; Shaheen et al., 2013). Salinity induced reduction in LA as well as the biomass can be interpreted as adaptive mechanisms to high salinity in *S. persica*. It has been reported that the reduction in LA produces an indirect benefit, because plants can thus limit water loss by transpiration, which in turn can favor the retention of toxic ions in roots, limiting the accumulation of these ions in the aerial part of the plant (Munns and Tester, 2008; Acosta-Motos et al., 2015).The RWC% of leaf remained unchanged at all levels of salinity in *S. persica*. On the contrary, severe reduction in RWC% are reported in many glycophytic plants imposed to salt stress (Kholova et al., 2009; D’Souza and Devaraj, 2010; Akçay et al., 2012; Shaheen et al., 2013).The growth reduction in *S. persica* at higher salinity might be an adaptive mechanism to survive under prolonged high salinity condition. The growth reduction can save energy cost, reduce ROS production, decrease amino acid demand for protein synthesis, and thereby provide more free amino acids for osmotic adjustment (Sobhanian et al., 2010; Yu et al., 2011).

Our results showed that Na^+^ level was continuously enriched in *S. persica* leaves with increasing salt concentration, but at 1000 mM NaCl, the amount of Na^+^ was surprisingly dropped down to the level of 250 mM NaCl treated plants (**Table 1**). In contrasts to our results there are several reports of gradual increase in leaf Na^+^ content with increase in salinity in other halophytes (Parida et al., 2004a; Qiu-Fang et al., 2005; Redondo-Gómez et al., 2007; Parida and Jha, 2010b; Sekmen et al., 2012; Takagi and Yamada, 2013; Theerawitaya et al., 2015). The observed decrease in the Na^+^ content in *S. persica* at extreme salinity may be due to the restriction of Na^+^ uptake and/or transport from roots to shoots (Teakle et al., 2013). In addition, a reduced transpiration rate may have restricted Na^+^ transport to the shoots (Abideen et al., 2014). K^+^ is commonly regarded as a most important cationic osmolyte and Ca^2+^ act as the membrane stabilizer or affects the capability of biomembranes to selectively absorb some ions (Basu et al., 2010; Parida and Jha, 2013). Our data showed that K^+^ content of leaf remained unaffected by salinity. Our results contrasts with several reports of decrease in K^+^ content in many halophytes (Redondo-Gómez et al., 2010; Yu et al., 2011; Takagi and Yamada, 2013) as well glycophytes (Shaheen et al., 2013; Ben Rejeb et al., 2015). These results suggest that the absorption and transportation of K^+^ to leaf tissue is not impaired by high salinity in leaf tissue of *S. persica*. The Ca^2+^ content of leaf did not change significantly at low, moderate and high salinity and dramatically decreased at extreme salinity in *S. persica*. The decrease in Ca^2+^ at extreme salinity may be due to salinity dominated by Na^+^ salts reduces Ca^2+^ availability and Ca^2+^ transport to growing regions of the plants (Hosseini et al., 2010). The unchanged level of Ca^2+^ content of leaf at low salinity suggests the role of this cation in protection of membrane. The reduction in plant total N concentration in extreme salinity (1000 mM NaCl) may be due to an increasing fraction of the N would be diverted to production of glycinebetaine and other ammonium compunds as the osmolytes (Redondo-Gómez et al., 2010). All the micronutrient levels except Cu^2+^ content remained unchanged in *S.persica*. The lack of interaction between Fe^2+^, Mn^2+^, and Zn^2+^ levels and salinity indicates that these important cations are not disturbed by salt stress in *S. persica*. The decline in Cu^2+^ by salinity may be due to salinity effects on the availability of this micronutrient, competitive uptake and transport, or partitioning within the plant organs. The photosynthetic pigments such as chlorophylls and carotenoids are vital components of energy metabolism in plants (Parida and Jha, 2013). The alterations in chlorophylls and carotenoids affects the plant metabolism significantly. In *S. persica*, chlorophyll and carotenoid contents remained unaffected by salinity. The unchanged level of chlorophyll pigments suggest that chlorophyllase activity does not change by salinity in *S. persica* thereby preventing the degradation of chlorophyll. In contrasts to our results, the significant reduction in chlorophyll and carotenoids has been reported in many halophytes such as *Arthrocnemum macrostachyum* (Redondo-Gómez et al., 2010), *Salicornia europaea* (Fan et al., 2011), *Sueda salsa*, and *Kochia scoparia* (Takagi and Yamada, 2013), *Panicum turgidum* (Koyro et al., 2013) and Quinoa (Amjad et al., 2015) as well as in glycophytes (Kholova et al., 2009; Shobbar et al., 2012; Boriboonkaset et al., 2013; Ben Rejeb et al., 2015). The excited triplet state of chlorophyll and singlet oxygen is quenched by the carotenoid and it also stabilizes and protect the lipid phase of the thylakoid membrane (Ramel et al., 2012; Parida and Jha, 2013). Our results suggest that coordinated changes in antioxidative enzymes scavenge the salinity induced ROS production and thereby protecting the pigments in the thylakoid membrane in *S. persica*.

The factors that causes reduction of photosynthesis in plant under stress conditions can be grouped into two categories, viz. stomatal limitation and non-stomatal limitation. The stomatal limitation refers to the decrease in CO_2_ diffusion through the stomata to the fixation site and non-stomatal limitation refers to the metabolic or biochemical capacity of leaves to fix CO_2_ (Fan et al., 2011). The decreased in intercellular CO_2_ levels may be the consequence of declined in stomatal conductance that led to the decrease in photosynthesis (Yu et al., 2011). In *S. persica*, photosynthesis (P_N_), stomatal conductance (g_s_), and transpiration (E) declined at higher (750 mM NaCl) and extreme (1000 mM) salinities, however, intercellular CO_2_ (C_i_) and WUE remained unaffected at all levels of salinity. The reduced photosynthesis observed did not lead to a reduction of the intercellular CO_2_ concentration (Ci) in *S. persica* which suggest that reduction in P_N_ in this plant may be due to non-stomatal limitation. Koyro et al. (2013) reported that the decline in photosynthesis at this condition might be caused by a reduction of the carboxylation activity of photosynthesis rather than any effect on CO_2_ diffusion. On the contrary, decline in *P*_N_, *g*_s_, and *E* is accompanied by a decrease in *C*_i_ and increase in WUE has been reported in the halophyte *Arthrocnemum macrostachyum* (Redondo-Gómez et al., 2010) and *Puccinellia tenuiflora* (Yu et al., 2011). As compared to *S. persica*, there was a severe reduction in *P*_N_, *g*_s_, and *E* by salinity in the glycophytes (Nazar et al., 2011; Boriboonkaset et al., 2013). In *S. persica*, reduction in *g*_s_ at high salinity may be due to stomatal closure thereby preventing the plant from transpiration water lose as evidenced from unchanged levels of RWC% and WUE in this plant. It has been reported that reduction of the stomatal conductance and consequently transpiration is an adaptive measures in halophytes to cope with excess salt (Koyro, 2006; Shabala et al., 2012).

In plants, the PSII light-harvesting system comprises of several chlorophyll a/b binding proteins which perform two vital functions, the efficient collection of light energy for photosynthesis and the dissipation of excess excitation energy in regulated manner (Fan et al., 2011). The maximal quantum efficiency of PSII (Fv/Fm) is a useful indicator of the photoinhibition or stress-induced damage to the PSII (Maxwell and Johnson, 2000). In *S. persica*, Fv/Fm ratio did not change significantly with increasing salinity which indicates that the excitation energy capturing ability and efficiency of PSII remained unaffected by salinity in this plant. In the salt sensitive glycophytic plants, salt stress has a converse effects on chlorophyll fluorescence parameters, i.e., decrease in maximum photochemical efficiency (Fv/Fm) (Nazar et al., 2011; Boriboonkaset et al., 2013; Lee et al., 2013; Ben Rejeb et al., 2015). In contrasts to our results, Fv/Fm ratio increases under low salinity and decreases under high salinity in the halophyte *S. europaea* (Fan et al., 2011) and increases at all levels of salinity in *Arthrocnemum macrostachyum* (Redondo-Gómez et al., 2010). In *S. persica* the decline in relative quantum yield of PSII (ΦPSII) with increasing salinity paralleled with decreasing in net photosynthetic rate (P_N_). Our results are in agreement with several reports suggesting that there is usually a theoretical linear relationship between ΦPSII and P_N_ (Maxwell and Johnson, 2000; Theerawitaya et al., 2015). However, the NPQ remained unaffected by salinity in *S. persica*. On the contrary, ΦPSII and NPQ remained unchanged in response to salinity in the halophytes *A. macrostachyum* (Redondo-Gómez et al., 2010). NPQ is a photo-protective mechanism that protects pigments, lipids and proteins in the photosynthetic thylakoid membrane from oxidative damage due to ROS produced by triplet chlorophyll by thermal dissipation of excess energy (Azzabi et al., 2012). It has been reported that the photorespiration and cyclic electron transport are two physiological processes could be mechanisms to protect against excess radiation under high salinities in *Sarcocornia fruticosa* (Redondo-Gómez et al., 2006) and *A. macrostachyum* (Redondo-Gómez et al., 2010). In *S. persica*, these two physiological processes could be the appropriate mechanisms to protect the plant from excess radiation under high salinities, because, as in the case of *S. fruticosa* and *A. macrostachyum*, the relatively stable NPQ was observed across the salinity range. These results suggest that salinity does not cause an increase in thermal dissipation in PSII antennae. Therefore, the salt tolerance of *S. persica* is partly attributed to its capacity to maintain the integrity of PS II function. In *S. persica* the ETR, photochemical quenching (q_p_), and P_N_ declined by high and extreme salinity which suggests that reduced flow of electrons through the photosystems took place to prevent *S. persica* from over excitation of the photosynthetic reaction centers as suggested by Koyro et al. (2013).

In glycophytes, as well as in halophytes, a common mechanism exists for ROS production and toxicity, but detoxification mechanisms vary in response to salinity (Ozgur et al., 2013). To avoid excessive ROS accumulation, plants possess a complex antioxidant defense system including non-enzymatic systems, for example, carotenoids, ascorbic acid, and glutathione in addition to ROS-scavenging enzymes such as SOD, CAT, POX, APX, and GR to protect the cellular membranes and organelles from detrimental effects of ROS. SODs dismutate $O_{2}^{•-}$ into H_2_O_2_ and have been considered to act as the ‘first line of defense’ against oxidative stress in plants (Bose et al., 2014). Several metal complexes of SOD such as Mn, Fe, and Cu/Zn isoforms occur in different cell compartments such as the cell wall, cytoplasm, mitochondria, and chloroplasts (Miszalski et al., 1998; Mittova et al., 2000). Cu/ZnSODs are reported to occur within chloroplasts (Miszalski et al., 1998), in the cytosol (Sakamoto et al., 1995), and in mitochondria (Miszalski et al., 1998). It has been reported that Fe-containing SOD located exclusively in chloroplasts (Miszalski et al., 1998). Mn-SODs are primarily located in mitochondria (Miszalski et al., 1998) and in peroxisomes (del Río et al., 1983; Miszalski et al., 1998), and their localization in chloroplasts has been also reported by some authors (Slooten et al., 1995). In plants, classical subcellular fractionation studies as well as *in situ* activity staining have established that the isoforms of CAT are predominantly localized in peroxisomes (Mhamdi et al., 2010). The different isoforms APX are localized in chloroplast, cytosol, mitochondria, and peroxisomes (Shigeoka et al., 2002; Caverzan et al., 2012). Several studies reported that the halophytes have efficient antioxidative defense system than the glycophytes (Seckin et al., 2010; Ellouzi et al., 2011; Srivastava et al., 2015). It has been reported that obstruction in photosynthesis would increase the production of ROS in cells, which can cause oxidative damage to membrane lipids, proteins, and DNA, thereby affecting the integrity of cellular membranes, enzyme activities, and the function of photosynthetic apparatus (Yu et al., 2011). However, in *S. persica*, high salinity induced impairment in photosynthesis did not cause, a significant increase in ROS levels and oxidative damage to the plant. Our results showed that the constitutive levels of all the antioxidative enzymes in the halophyte *S. persica* was much higher than that reported in glycophytes (Lee et al., 2001, 2013; Oztiirk and Demir, 2003; Nazar et al., 2011; Ben Rejeb et al., 2015).Therefore, the tolerance to high salinity in *S. persica* derives largely from the constitutively maintained higher antioxidative enzymatic activities.

Membrane lipid peroxidation is a useful indicator of free radical formation in plants exposed to adverse environmental conditions such as salinity and drought (Parida and Jha, 2013). The lipid peroxidation level measured in terms of MDA content did not change significantly by salinity suggesting that the halophyte *S. persica* have an efficient antioxidative defense system to scavenge the production of ROS. It is logical because significant ROS accumulation (H_2_O_2_ and $O_{2}^{•-}$) has not been detected in *S. persica*. In contrasts to our results the lipid peroxidation level increase progressively by high salinity in many glycophytes such as mungbean (Nazar et al., 2011), rice (Shobbar et al., 2012), eggplants (Shaheen et al., 2013), and *Arabidopsis* (Ben Rejeb et al., 2015). Moreover, the activity and isoforms of various ROS scavenging enzymes was not consistently up-regulated in *S. persica*. The activity and isoforms of the vital antioxidative enzyme SOD which is the first line of defense against oxidative stress decreases in *S. persica* by salinity. On contrary, SOD activity increases consistently in many halophytes in response to salinity (Parida et al., 2004b; Amor et al., 2005; Benzarti et al., 2012; Duarte et al., 2013; Amjad et al., 2015). However, in *S. persica*, the SOD level is quite high in control plants (105 U mg^-1^ protein) than that reported in other halophytes (4–20 U mg^-1^ protein). These results suggest that the enzyme SOD is constitutively at a threshold level in *S. persica* to scavenge the salinity induced production of $O_{2}^{•-}$. A decrease in the SOD level at high salinity may be due to more utilization of the enzyme to sequester $O_{2}^{•-}$ radicals or due to the minimal synthesis of the SOD isoforms at a high external salt concentrations. In *S. persica*, the activity of CAT and the expression of its isoform increased at all levels of salinity, whereas the activity POX decreased. On the other hand, the activity of APX increased at low salinity (250–500 mM NaCl) and remained unchanged at high salinity (750–1000 mM NaCl). The increase activity of CAT might be involved in the detoxification of H_2_O_2_ in *S. persica*. It has been reported that the turnover rate of CAT is very high and in every second, one unit of CAT protein complex can decompose millions of molecules of H_2_O_2_ (Bose et al., 2014). In contrasts to our results the CAT activity has been reported to decrease or remained unchanged by high salinity in some halophytes (Parida et al., 2004b; Benzarti et al., 2012; Takagi and Yamada, 2013). The unchanged activity of APX at high salinity suggests that the APX constitutively present in this plant might be at a threshold level to detoxify the H_2_O_2_ in chloroplast and cytosol of *S. persica*. POXs are involved in many functions in plant cells such as ROS generation and regulation, H_2_O_2_ level regulation, oxidation of various substrates and also involved in loosening of the cross-linking of cell wall compounds (Passardi et al., 2005).The decrease activity of POX and down regulation of its isoform (predominantly POX1) might be involved in maintaining the appropriate level of H_2_O_2_ since H_2_O_2_ acts as a signaling molecule. However, there are several reports of salinity induced increase in both APX and POX in some halophytes (Parida et al., 2004b; Benzarti et al., 2012; Amjad et al., 2015) contrasts with our results. However, in the halophyte *Puccinellia tenuiflora*, both the enzyme activity remained stable under salinity (Yu et al., 2011). The GR is a redox regulatory enzyme like APX and it is essential for maintaining the redox-state of ascorbate and glutathione (Chalapathi Rao and Reddy, 2008; Benzarti et al., 2012). GR plays an important role in the control of endogenous H_2_O_2_ content through an oxido-reduction cycle (Halliwell-Asada pathway) involving glutathione and ascorbate (Noctor and Foyer, 1998; Bose et al., 2014). In *S. persica*, the activity of GR and its isoform was found to be downregulated by high salinity. On contrary, the GR level remains stable in some halophytes (Parida et al., 2004b; Yu et al., 2011) or increases in some halophytes (Benzarti et al., 2012) under salinity condition. Our results suggest that that the enzyme GR is at a threshold level in *S. persica* under high saline condition to maintain the redox-state of ascorbate and glutathione thereby protecting the plant from oxidative damage.

## Conclusion

**FIGURE 10:**
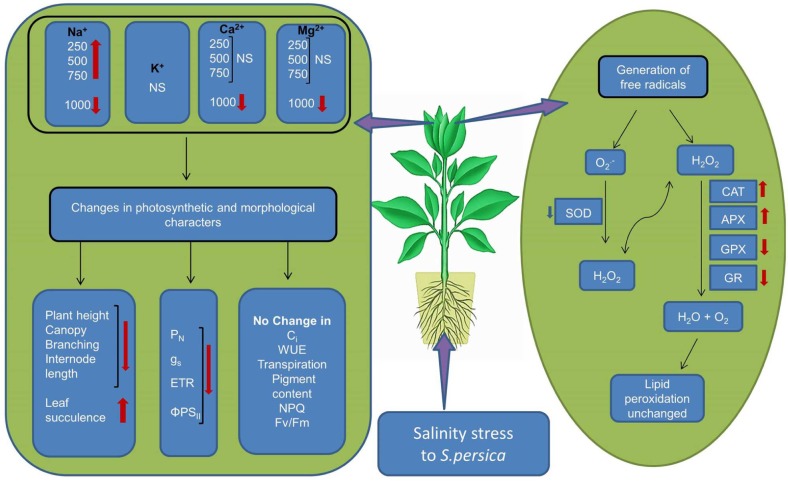
**Schematic representation of salinity induced changes in the halophyte *S. persica*.** ^∗^NS, No significant change.

The data presented in this work demonstrated that *S. persica* tolerate extreme hypersaline conditions by maintaining plant water status and nutrient uptake. The extreme saline condition has no deleterious effects on plant metabolism. The growth reduction at high salinity is an adaptive strategy in *S. persica* that can save energy cost, reduce ROS production, decrease amino acid demand for protein synthesis, and thereby provide more free amino acids for osmotic adjustment. The reduction in photosynthesis in *S. persica* at high salinity is due to non-stomatal limitation. This may be the cause of a reduction of the carboxylation activity of photosynthesis rather than any effect on CO_2_ diffusion. The integrity of the photosystem (PSII) component of the chloroplast is not adversely affected by extremely high salinity in *S. persica* as evidenced from the chlorophyll fluorescence data. Our results clearly indicate that high salinity induced production of $O_{2}^{•-}$ is scavenged by constitutively higher enzyme activity of SOD. Increased activity of SOD induces an overproduction H_2_O_2_ which is counter balanced by increased activity of CAT and high level of APX activity thereby maintaining an appropriate levels of H_2_O_2_. The coordinated changes in the activity of various antioxidative enzymes efficiently scavenge salinity induced ROS production thereby protecting the membrane integrity as evidenced from unchanged level of membrane lipid peroxidation in *S. persica*.Our data strongly propose that induction of antioxidant defenses by high level of antioxidative enzymes, is at least one component of the tolerance of *S. persica* to long-term salinity as evidenced by the growth behavior of the plants (**Figure 10**). Our data revealed that sustainable utilization of *S. persica* as a genetic resource can lead to develop salt tolerant crops by genetic engineering or breeding strategies.

## Author Contributions

JR performed most of the experiments. AKP designed and coordinated the experiments, analyzed the data, interpreted the results, and improved the manuscript. AP conducted some experiments and prepared the manuscript. AK maintained the plants, prepared the media and reagents, gave periodical stress treatments, and performed some experiments.

## Conflict of Interest Statement

The authors declare that the research was conducted in the absence of any commercial or financial relationships that could be construed as a potential conflict of interest.
